# The Correlation of Central Serous Chorioretinopathy and Subsequent Cardiovascular Diseases of Different Types: A Population-Based Cohort Study

**DOI:** 10.3390/ijerph16245099

**Published:** 2019-12-13

**Authors:** Hung-Jui Hsu, Chia-Yi Lee, Shih-Chun Chao, Chan-Wei Nien, Shih-Hao Tzeng, Jing-Yang Huang, Tai-Chuan Ko, Shun-Fa Yang, Hung-Yu Lin

**Affiliations:** 1 Institute of Medicine, Chung Shan Medical University, Taichung 402, Taiwan; juihee@hotmail.com (H.-J.H.); ysf@csmu.edu.tw (S.-F.Y.); 2 Department of Ophthalmology, Show Chwan Memorial Hospital, Changhua 500, Taiwan; ao6u.3msn@hotmail.com (C.-Y.L.); arthurking2727@yahoo.com.tw (S.-C.C.); skylightwave@hotmail.com (C.-W.N.); CMghost325@gmail.com (S.-H.T.); 3 Department of Optometry, College of Medicine and Life Science, Chung Hwa University of Medical Technology, Tainan 717, Taiwan; 4 Department of Electrical and Computer Engineering, National Chiao Tung University, Hsinchu 300, Taiwan; 5 Department of Optometry, Central Taiwan University of Science and Technology, Taichung 406, Taiwan; 6 Department of Ophthalmology, Chang Gung Memorial Hospital, Linkou 333, Taiwan; 7 Department of Medical Research, Chung Shan Medical University Hospital, Taichung 402, Taiwan; wchinyang@gmail.com; 8 Department of Optometry, Jen-Teh Junior College of Medicine, Nursing and Management, Miaoli 356, Taiwan; Kcc33546@gmail.com; 9 Department of Optometry, Chung Shan Medical University, Taichung 402, Taiwan; 10 Department of Exercise and Health Promotion, Chung Chou University of Science and Technology, Changhua 510, Taiwan

**Keywords:** central serous chorioretinopathy, cardiovascular disease, atherosclerosis, severity, epidemiology

## Abstract

The aim of the present study was to survey the relationship between central serous chorioretinopathy (CSC) and several cardiovascular diseases (CVDs) with different severities using the National Health Insurance Research Database. A retrospective cohort study was conducted. Subjects with CSC were enrolled according to the relevant diagnostic codes, and an age- and gender-matched population was used as the control group with a 1:4 ratio. The main outcome being considered was the development of CVD after CSC exposure. Cox proportional hazard regression was applied to calculate the adjusted hazard ratio (aHR) of CSC and CVD of different types. A total of 2865 patients that were diagnosed with CSC were enrolled in the study group, while another 11,460 non-CSC subjects were selected as the control group. There were 171 events of CVD which occurred in the study group, while another 557 cases were found in the control group. No significant differences were observed among the CVD cases between the study and control group, whether they had an acute or chronic form, according to the aHR. In the subgroup analysis, there was a significantly higher risk of CVD development in the male population aged from 40 to 59 years (aHR: 1.351, confidence interval (CI): 1.063–1.716), which was mainly due to the higher risk of mild CVD (aHR: 1.391, CI: 1.062–1.822). On the contrary, there was no significant difference in CVD development in any of the age subgroups of the female population. In conclusion, the existence of CSC is correlated with a higher rate of chronic CVD occurrence in the middle-aged male population.

## 1. Introduction

Cardiovascular diseases (CVDs) are the single most common cause of death from non-communicable diseases globally, and about 17.6 million individuals died of CVDs worldwide in 2012 [1]. In the Asian population, a higher risk of mortality has been observed compared to other ethnicities [2,3,4], which may result from the rapid urbanization process and the absence of related health policies [4]. There are different subtypes of CVDs, which include myocardial infarction (MI), acute coronary syndrome (ACS), coronary heart disease, and atherosclerosis [5]. If left untreated, the progression of atherosclerosis can eventually lead to the development of arterial wall rupture and MI [6].

Several studies have illustrated the relationship between CVD and ocular diseases involving retinal vessel occlusions, cataracts, and age-related macular degeneration [7]. The presence of CVD can contribute to retinal vessel occlusion, including the ischemic subtype that can lead to blindness [8,9]. CVD is also a risk factor for the occurrence of age-related macular degeneration and ocular ischemic syndrome [10,11]. On the other hand, the time sequence between CVD and ocular diseases like retinal vessel occlusion can be reversed. For instance, retinal vein occlusion is associated with a two-fold increased risk of MI development in the male population, according to a previous epidemiological study [12].

Central serous chorioretinopathy (CSC) features pigment epithelial detachment and serous retinal detachment, which mainly occur in the macular area [13]. Regarding the relationship between CSC and CVD, elevated plasminogen activator inhibitor 1 has been observed in both CSC and CVD, which indicates that a coagulation imbalance occurs in both diseases [14,15]. In addition, CSC and CVD tend to develop in males [1,13]. A previous study illustrated that coronary heart disease is not a risk factor for CSC [16], while another two studies suggested that the presence of CSC is related to the development of CVD, especially in the male population [17,18]. Thus, the possible relationship between CSC and CVD should be further investigated. Additionally, previous studies did not evaluate the association between CSC and the different types of CVD in the same population.

The aim of the current study was to evaluate the correlation between CSC and CVD of different severities via the application of the National Health Insurance Research Database (NHIRD) of Taiwan. In addition, the effects of CSC on CVD in different genders were also analyzed.

## 2. Materials and Methods

### 2.1. Data Source

This retrospective, population-based cohort study was approved by both the Institutional Review Board of Chung Shan Medical University (Project identification code: CS-17075) and the National Health Insurance Administration. Additionally, the current study adhered to the declaration of Helsinki in 1964 and its late amendment. The NHIRD contains insurance data from nearly all Taiwanese people, which was provided by the National Health Insurance Administration. The claims data were collected from the Longitudinal Health Insurance Database 2005 version (LHID 2005), which included documents on two million patients who were randomly sampled from the NHIRD institution in 2005 and were linked from 1 January 2000 to 31 December 2016. The International Classification of Diseases, Ninth Revision (ICD-9) and International Classification of Diseases, Tenth Revision (ICD-10) were used for diagnosis of diseases in the database. In addition, tbasic demographics, income level, and living region were also available from LHID 2005 and NHIRD.

### 2.2. Subject Selection

Subjects were defined as having CSC if the claimed data indicated (1) receipt of the diagnostic code of CSC, (2) the arrangement of optical coherence tomography, and (3) receipt of the CSC diagnosis by an ophthalmologist. To better evaluate the potential association between CSC and CVD, the following exclusion criteria were used to exclude some statuses: (1) diagnosed with legal blindness at any time, to exclude those without vision; (2) diagnosed with ocular tumors before the index date, to exclude those with severe ocular damage; (3) arrangement of eyeball removal surgery before the index date, to exclude those with severe ocular damage; (4) diagnosed with CVD (diagnostic codes are shown in the following section), age-related macular degeneration, or retinal vessel occlusion before the index date, to exclude those who developed a primary outcome before the index date or with ocular diseases that may be related to CSC; (5) aged younger than 20 or older than 100, to standardize the age distribution; and (6) diagnosis of CSC was earlier than 2005, to standardize the disease interval of CSC. For the comparison, every subject in the study group was age- and gender-matched with four patients without CSC, and this population served as the control group. Moreover, patients were excluded from the current study if they could not be matched with the four non-CSC individuals.

### 2.3. Main Outcome Measurement

The presence of CVD, including MI, ACS, chronic ischemic heart disease, and atherosclerosis, was defined as the primary outcome in the current study according to: (1) the emergence of CVD-related ICD-9/ICD-10 diagnostic codes one year after the index date; (2) the receipt of an electrocardiogram before CVD diagnosis; and (3) the receipt of a blood test, including a complete blood cell count and lipid profiles, before CVD diagnosis. Since hypertension may be asymptomatic in a large number of individuals, which would mean they do not visit the hospital, leading to significant underestimation of the case numbers, hypertension was only selected as a covariate rather than the primary outcome in the current study. In addition, only individuals that received CVD diagnostic codes from an internal physician were recognized as having achieved the primary outcome and were included in the current study.

### 2.4. Demographic Variables and Co-Morbidities

To ensure the general conditions of each subject were as similar as possible, the influence of the following parameters was also evaluated in the multivariate analysis model of the current study: age, gender, education level, marriage status, hypertension, diabetes mellitus, hyperlipidemia, peripheral vascular disease, chronic pulmonary disease, rheumatic disease, peptic ulcer disease, and kidney disease. We traced the data in the LHID 2005 longitudinally from the index date of each subject to (1) the date of CVD diagnosis, (2) participant withdrawal from the National Health Insurance program, or (3) the end date of NHIRD, which was 31 December 2016.

### 2.5. Statistical Analysis

SAS version 9.4 (SAS Institute Inc., Cary, NC, USA) was used for the analyses mentioned in the current study, and the statistical methods were similar to those used in previous studies [19]. After age- and gender-matching with 1:4 proportions of both the study group and control group, a Poisson regression was performed to calculate the incidence rate of CVD and 95% confidence intervals (CI). In the next step, we used multiple Cox proportional hazard regressions to produce the adjusted hazard ratios (aHR) of CVD in the CSC population compared to the non-CSC group, by combining all the demographic information as well as systemic co-morbidities in the analysis model to survey whether CSC is an independent risk factor for subsequent CVD. In the Cox proportional hazard regression, the hazard ratio of each potential risk factor (i.e., demographic information and systemic co-morbidities) was calculated, then the impact of each potential risk factor on CVD was neutralized to erase the effects from those confounders and investigate the association between CSC and CVD more precisely. To evaluate the effect of CSC on CVD of different types, the CVD was divided into acute CVD (MI and ACS) and chronic CVD (chronic ischemic heart disease and atherosclerosis). Following this, the influences of SCS on the development of the two CVD subgroups/subtypes were investigated separately. On the other hand, a subgroup analysis according to the age and gender of participants in the study group was also conducted. We presented Kaplan–Meier curves to demonstrate the cumulative incidence probability of CVD with different severities between the study and control groups, and then conducted the log-rank test to evaluate whether a significant difference existed between the two survival curves. Since almost all subjects in the NHIRD are from the Han/Chinese population, ethnicity was not considered as a confounding factor in the current study. Statistical significance was regarded as a *p*-value less than 0.05. Due to the calculation method in the statistical software, a *p*-value less than 0.0001 was described as *p* < 0.0001.

## 3. Results

**Figure 1:**
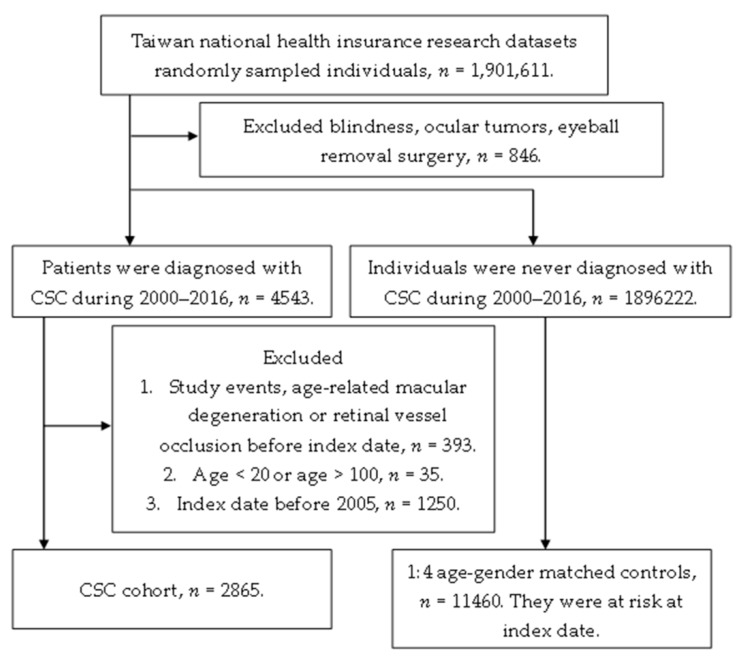
The flowchart of patient selection. CSC: central serous chorioretinopathy.

A total of 2865 patients diagnosed with CSC were enrolled in the study group, while another 11,460 non-CSC subjects were selected as the control group. The flowchart of the selection is shown in Figure 1. Due to the matching process, the age and gender distributions are identical between the study and the control groups, while the other demographic data and systemic co-morbidities are revealed in Table 1. There were higher education levels and marriage status in the patients with CSC, and the systemic comorbidities were all significantly higher in the study group with the exception of peripheral vascular disease and kidney disease.

**Figure 2:**
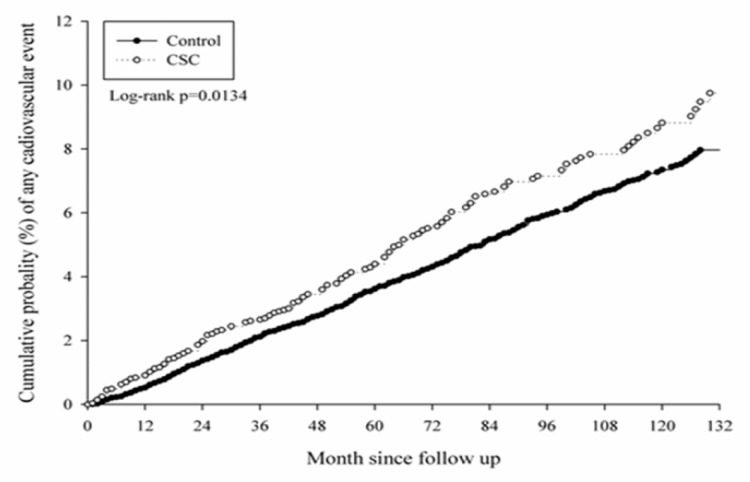
Kaplan–Meier curve of all cardiovascular diseases in the middle-aged male population.

**Figure 3:**
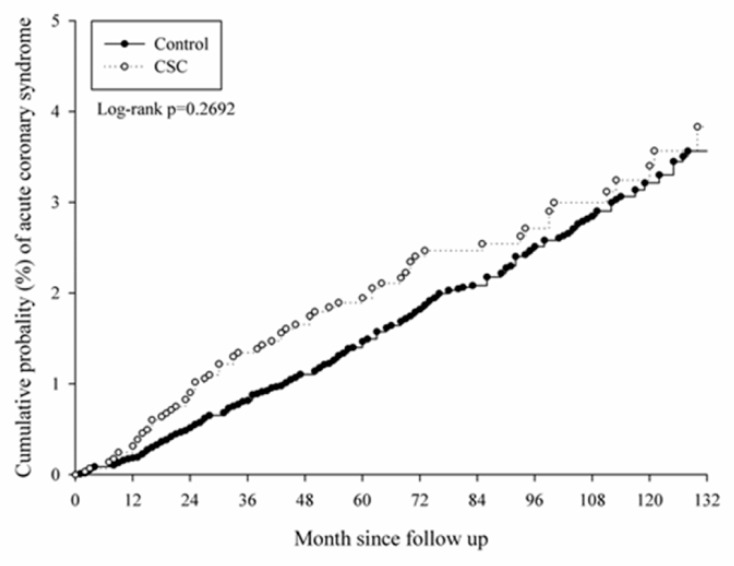
Kaplan–Meier curve of acute cardiovascular diseases in the middle-aged male population.

**Figure 4:**
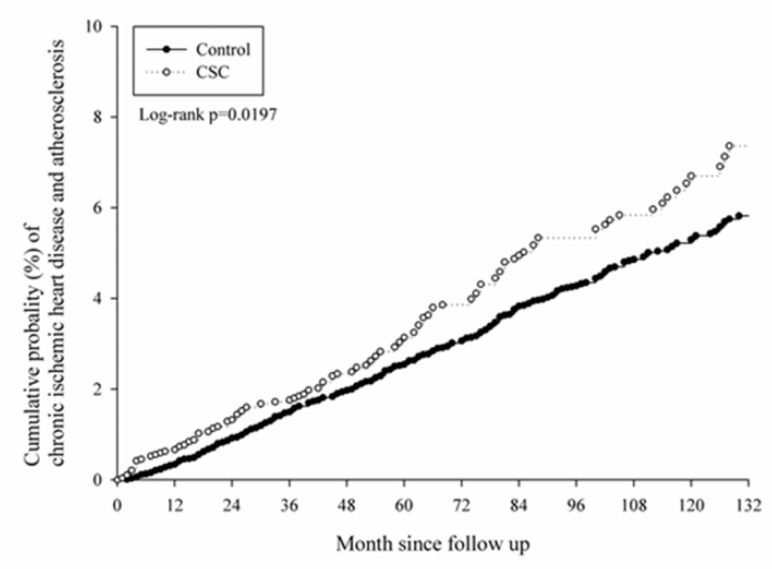
Kaplan–Meier curve of chronic cardiovascular diseases in the middle-aged male population.

After a follow-up interval of up to 16 years, there were 171 events of CVD that occurred in the study group, while another 557 cases were found in the control group. No significant differences were observed in the CVD cases, whether acute or chronicform, according to the aHR in the Cox proportional hazard regression (Table 2). In the subgroup analysis, there was a significantly higher risk of CVD development in the male population aged from 40 to 59 (aHR: 1.351, CI: 1.063–1.716), which was mainly due to the higher risk of chronic CVD (aHR: 1.391, CI: 1.062–1.822) (Table 3). On the contrary, there was no significant difference in CVD development in all the age subgroups of the female population (Table 4). The cumulative probability of CVD in males with CSC and aged from 40 to 59 is shown in Figure 2, Figure 3 and Figure 4.

## 4. Discussion

Briefly, the current study demonstrates that CSC did not lead to a prominent risk of CVD development in the gross population. However, males with CSC and aged from 40 to 59 years old demonstrated a significantly higher risk of CVD, mainly resulting from the elevation probability of chronic CVD. The female population with CSC did not show a prominent risk of CVD in any of the age subgroups.

Although they have not been fully elucidated, there are several pathways that have been proposed for the development of CSC [20]. Aldosterone dysregulation is a possible route for CSC occurrence which has been established before, through the mineralocorticoid receptors located in the kidneys, neurosensory retina, and choroid [20]. The primary function of aldosterone is to export excessive fluid and sodium to maintain body homeostasis via the activation of mineralocorticoid receptors [21]. If the aldosterone pathway was altered, fluid would be retained in the body and lead to congestive heart failure and secondary hypertension [22,23], which could occur in the choroidal vascular structure in patients with retinal venous occlusion and CSC via the inappropriate activation of mineralocorticoid receptors and related choroidal vasodilation [13,24]. In addition to the fluid-sodium pathway, elevated platelet aggregation and hypercoagulability were also found in patients with CSC in previous research [25]. Moreover, an inflammatory process can be observed in patients with CSC, in which elevated cytokines, including vascular endothelial growth factor and interleukin-8, can be found in the aqueous humor [26]. Concerning the mechanism of CVD, renal impairment with sodium retention, like in those with primary aldosteronism, is associated with CVD development or progression, including coronary artery disease and left ventricular hypertrophy [27]. Further, hypercoagulability is correlated with thrombosis formation and atherosclerosis [28]. Atherosclerosis, as well as coronary artery disease, results from the inflammation reaction, which leads to macrophage aggregation and formation of plaque [29]. Moreover, optical coherence tomography angiography revealed a prominent choroidal flow void area in CSC [30], which is similar to the ischemic changes of CVD. Consequently, CSC and CVD share similar etiologies, and CSC may be an early sign of such pathophysiologies in the fine retinal vessels, while the large vessels in the circular system are influenced later. This pathway is supported by the findings of the current study.

The relationship between CSC and CVD has been evaluated in previous studies, but without firm consensus. In two previous studies conducted in the same region as the current study, the presence of CSC was found to be significantly related to coronary heart disease and ischemic stroke [17,18], while another study indicated that CVD is not a risk factor of CSC if the multivariable analysis considers several potential risk factors like age, male gender, hypertension, and smoking [16]. In the current study, the gross aHR of CHD did not show a significant difference between the study and the control groups, which may support the findings of Chatziralli et al. that the gross relationship between CSC and CVD is not significant. Nevertheless, the middle-aged male population showed a significantly higher rate of chronic ischemic heart disease and atherosclerosis occurrence compared to those non-CSC individuals at a similar age interval, and the probability of CVD was correlated to the disease period of CSC, which has rarely been demonstrated elsewhere. Comparing our results to those in the study written by Chen et al., both results revealed the significant correlation between CSC and CVD in the middle-aged male population (40 to 59 years old in the current study and 20 to 64 years old in the study written by Chen et al.) [17]. There are two potential explanations for this phenomenon. First, the middle-aged male population has the highest rate of CSC development and the severity of CSC is also the highest in this population, which may lead to a higher rate of CVD development via the three pathways that discussed earlier. Furthermore, the age for seeking a health examination is often around 40 years old in Taiwan, thus CVD could be found more frequently around this age than in the younger population. Still, the current study found that the effect of CSC is mainly on those with chronic CVD, while the study written by Chen et al. did not find this.

CVD impacts 7.6% and 5.0% of the male and female population in the USA, respectively [31], and has remained the leading cause of death in recent years [1,31,32]. Despite the fatal effect of these diseases, MI and ACS are derived from the progression of chronic CVD, like chronic ischemic heart disease and atherosclerosis [6,33]. In the current study, the presence of CSC was found to lead to the development of chronic ischemic heart disease and atherosclerosis significantly more often in the middle-aged male population, which may progress to MI if a longer disease period occurs. Moreover, the percentage of hypertension and hyperlipidemia, both of which are risk factors for acute MI [34], was also significantly higher in patients with CSC in the current study. Accordingly, patients with CSC should be referred to internal medicine departments for possible CVD evaluation.

There are still some limitations in the current study. First, the retrospective and observational design diminishes the homogeneity between the study and control groups. In addition, the disease course of both CSC and CVD cannot be evaluated, thus the accuracy of the causal relationship may be retarded. Additionally, smoking is a prominent risk factor for CVD [35], but we did not include this confounder in the multivariable analysis because the ICD system does not have a specific code for cigarette smoking, therefore physicians in Taiwan rarely register their patients’ personal histories, including smoking, alcohol consumption, and betel/areca nut chewing, into the claimed database. Moreover, obesity is also a prominent risk factor of CVD, but we could not include it in the multivariable analysis due to the infrequent use of the related diagnostic codes in Taiwan (<0.001% according to our experience) except in some bariatric surgery cases. In clinical practice, physicians prefer to enter obesity-associated diseases like diabetes mellitus or hyperlipidemia into the insurance system. Still, we included some diseases related to obesity, including hypertension, hyperlipidemia, and diabetes mellitus. As a result, the influence of not including obesity as a confounder might be reduced.

## 5. Conclusions

In conclusion, the presence of CSC is correlated to a higher rate of chronic CVD development in the middle-aged male population. As a consequence, routine examination for CVD is recommended for this population, in order to reveal potential cardiovascular events and arrange proper management. Further large-scale prospective studies to evaluate whether the existence of CSC alters the prognosis of different types of CVD are also needed.

## Figures and Tables

**Table 1:** Baseline characteristics.

	Study	Control	*p*-Value
N	2865	11,460	
Age			1.0000
<40	1189 (41.5%)	4756 (41.5%)	
40–59	1426 (49.77%)	5704 (49.77%)	
≥60	250 (8.73%)	1000 (8.73%)	
Sex			1.0000
Male	1899 (66.28%)	7596 (66.28%)	
Female	966 (33.72%)	3864 (33.72%)	
Education			0.0006
<6	390 (13.61%)	1754 (15.31%)	
6–9	578 (20.17%)	2519 (21.98%)	
9–12	1466 (51.17%)	5723 (49.94%)	
≥12	431 (15.04%)	1464 (12.77%)	
Marriage			0.0014
Not married	931 (32.5%)	3921 (34.21%)	
Married	1739 (60.7%)	6651 (58.04%)	
Co-morbidities			
Hypertension	316 (11.03%)	988 (8.62%)	<0.0001
Diabetes mellitus	162 (5.65%)	494 (4.31%)	0.0021
Hyperlipidemia	194 (6.77%)	584 (5.1%)	0.0004
Peripheral vascular disease	8 (0.28%)	20 (0.17%)	0.2564
Chronic pulmonary diseases	73 (2.55%)	218 (1.9%)	0.0284
Rheumatic disease	19 (0.66%)	32 (0.28%)	0.0020
Peptic ulcer disease	126 (4.4%)	388 (3.39%)	0.0092

**Table 2:** Study events and risk in the study and control groups.

	Study			Control			aHR (95% CI)
Event	Person Months	Event	Rate * (95% CI)	Person Months	Event	Rate * (95% CI)	
All CVD	217,982	171	7.84 (6.75–9.11)	880,624	557	6.33 (5.82–6.87)	1.124 (0.935–1.350)
Acute CVD	223,320	69	3.09 (2.44–3.91)	895,898	238	2.66 (2.34–3.02)	1.073 (0.807–1.426)
Chronic CVD	220,087	126	5.72 (4.81–6.82)	887,702	401	4.52 (4.10–4.98)	1.120 (0.903–1.389)

CVD: cardiovascular disease; * Incidence rate of cardiovascular events per 10,000 person months. aHR: adjusted hazard ratio, the considered co-variates included age, gender, education, marriage status, and co-morbidities. CI: confidence interval.

**Table 3:** Study events in the male population from the subgroup analysis.

Event	Rate *		aHR (95% CI)
	Study	Control	
Aged <40			
All CVD	2.43 (1.44–4.10)	3.40 (2.72–4.24)	0.625 (0.351–1.112)
Acute CVD	1.21 (0.58–2.54)	1.86 (1.38–2.51)	0.550 (0.245–1.233)
Chronic CVD	1.73 (0.93–3.22)	2.08 (1.57–2.76)	0.753 (0.377–1.501)
Aged 40–59			
All CVD	12.63 (10.31–15.48)	8.49 (7.52–9.60)	1.351 (1.063–1.716)
Acute CVD	4.41 (3.15–6.17)	3.38 (2.79–4.09)	1.252 (0.848–1.847)
Chronic CVD	9.93 (7.91–12.47)	6.39 (5.56–7.35)	1.391 (1.062–1.822)
Aged ≥60			
All CVD	22.13 (13.94–35.12)	20.65 (16.31–26.15)	0.962 (0.565–1.637)
Acute CVD	8.06 (3.84–16.90)	7.07 (4.78–10.47)	0.889 (0.375–2.104)
Chronic CVD	14.07 (7.99–24.77)	15.94 (12.21–20.81)	0.764 (0.403–1.449)

CVD: cardiovascular disease. * Incidence rate per 10,000 person months. aHR: adjusted hazard ratio, the considered co-variates included age, gender, education, marriage status, and co-morbidities. CI: confidence interval.

**Table 4:** Study events in the female population from the subgroup analysis.

Event	Rate *		aHR (95% CI)
	Study	Control	
Aged <40			
All CVD	1.92 (0.96–3.85)	1.14 (0.73–1.78)	1.798 (0.759–4.260)
Acute CVD	1.20 (0.50–2.88)	0.54 (0.28–1.03)	2.508 (0.813–7.731)
Chronic CVD	0.72 (0.23–2.23)	0.60 (0.32–1.11)	1.236 (0.311–4.911)
Aged 40–59			
All CVD	7.24 (4.77–11.00)	6.54 (5.25–8.16)	0.934 (0.575–1.518)
Acute CVD	2.26 (1.08–4.75)	3.00 (2.17–4.14)	0.665 (0.290–1.526)
Chronic CVD	5.22 (3.20–8.52)	4.34 (3.31–5.67)	1.012 (0.569–1.802)
Aged≥60			
All CVD	19.12 (10.59–34.52)	20.47 (15.33–27.33)	0.926 (0.473–1.813)
Acute CVD	10.02 (4.50–22.31)	7.57 (4.77–12.01)	1.030 (0.381–2.784)
Chronic CVD	11.75 (5.60–24.64)	13.90 (9.83–19.65)	0.873 (0.379–2.012)

CVD: cardiovascular disease. * Incidence rate per 10,000 person months. aHR: adjusted hazard ratio, the considered co-variates included age, gender, education, marriage status, and co-morbidities. CI: confidence interval.
